# Inhibition of IκB Kinase Is a Potential Therapeutic Strategy to Circumvent Resistance to Epidermal Growth Factor Receptor Inhibition in Triple-Negative Breast Cancer Cells

**DOI:** 10.3390/cancers14215215

**Published:** 2022-10-24

**Authors:** Yong Weon Yi, Kyu Sic You, Sanghee Han, In Jin Ha, Jeong-Soo Park, Seok-Geun Lee, Yeon-Sun Seong

**Affiliations:** 1 Department of Biochemistry, College of Medicine, Dankook University, Cheonan 31116, Chungcheongnam-do, Korea; 2 Graduate School of Convergence Medical Science, Dankook University, Cheonan 31116, Chungcheongnam-do, Korea; 3 Graduate School, Kyung Hee University, Seoul 02447, Korea

**Keywords:** anticancer, combination, gefitinib, epidermal growth factor receptor (EGFR) inhibition, IκB kinase (IKK) inhibition, kinase inhibitor, resistance, synergism, triple-negative breast cancer (TNBC)

## Abstract

**Simple Summary:**

Triple-negative breast cancer (TNBC) is an aggressive and intractable malignancy. Although a high-level expression of epidermal growth factor receptor (EGFR) is a distinct feature of TNBC, targeting EGFR has not been successful yet. Here, we described a combination of the EGFR inhibitor gefitinib and IκB kinase (IKK) inhibitor IKK16 (Gefitinib+IKK16) as a potential therapeutic approach to treat TNBC. The combination of these drugs resulted in reduced cell viability and survival in TNBC cells in vitro. Mechanistically, several components of PI3K/AKT/mTOR pathway were further downregulated by drug combination compared with single-agent treatments. Gene expression analysis revealed that several NF-κB/RELA targets were suppressed, and a couple of tumor suppressor genes were induced by the drug combination. Taken together, targeting IKK may potentiate EGFR inhibition in TNBC.

**Abstract:**

Triple-negative breast cancer (TNBC) remains as an intractable malignancy with limited therapeutic targets. High expression of epidermal growth factor receptor (EGFR) has been associated with a poor prognosis of TNBC; however, EGFR targeting has failed with unfavorable clinical outcomes. Here, we performed a combinatorial screening of fifty-five protein kinase inhibitors with the EGFR inhibitor gefitinib in the TNBC cell line MDA-MB-231 and identified the IκB kinase (IKK) inhibitor IKK16 as a sensitizer of gefitinib. Cell viability and clonogenic survival assays were performed to evaluate the antiproliferative effects of the gefitinib and IKK16 (Gefitinib + IKK16) combination in TNBC cell lines. Western blot analyses were also performed to reveal the potential mode of action of this combination. In addition, next-generation sequencing (NGS) analysis was performed in Gefitinib+IKK16-treated cells. The Gefitinib+IKK16 treatment synergistically reduced cell viability and colony formation of TNBC cell lines such as HS578T, MDA-MB-231, and MDA-MB-468. This combination downregulated p-STAT3, p-AKT, p-mTOR, p-GSK3β, and p-RPS6. In addition, p-NF-κB and the total NF-κB were also regulated by this combination. Furthermore, NGS analysis revealed that NF-κB/RELA targets including CCL2, CXCL8, EDN1, IL-1β, IL-6, and SERPINE1 were further reduced and several potential tumor suppressors, such as FABP3, FADS2, FDFT1, SEMA6A, and PCK2, were synergistically induced by the Gefitinib-+IKK16 treatment. Taken together, we identified the IKK/NF-κB pathway as a potential target in combination of EGFR inhibition for treating TNBC.

## 1. Introduction

Triple-negative breast cancer (TNBC) is immunohistochemically negative for both the estrogen receptor (ER) and progesterone receptor (PR) and exhibits no amplification of the human epidermal growth factor receptor 2 (HER2), either [1,2]. TNBC is the most aggressive subtype of breast cancers, accounting for approximately 20% of them [1,2,3,4,5,6]. Until recently, no option of targeted therapy was available. Currently, four targeted therapies against three targets are clinically available: (i) the poly [ADP-ribose] polymerase 1 (PARP1) inhibitors olaparib (Lynparza^®^) and talazoparib (Talzenna^®^); (ii) the programmed cell death ligand 1 (PD-L1) inhibitor atezolizumab (Tecentriq^®^); and (iii) the antibody drug conjugate (ADC) sacituzumab govitecan (Trodelvy^®^) [2]. Unfortunately, most treatments still demonstrate limited clinical outcomes [2]. Therefore, there is an unmet medical need to investigate therapeutic targets and develop novel therapeutic strategies for TNBC treatment.

The epidermal growth factor receptor (EGFR) is a ubiquitously expressed receptor tyrosine kinase (RTK) and serves as a therapeutic target to treat cancers, such as non-small cell lung cancer (NSCLC), metastatic colorectal cancer (mCRC), and advanced head and neck cancer (HNC), which exhibit upregulated EGFR activity [7,8]. Up to now, various EGFR inhibitors (EGFRis) have been approved as anticancer drugs including: (i) small-molecule EGFRis such as gefitinib (Iressa^®^), erlotinib (Tarceva^®^), lapatinib (Tykerb^®^), icotinib (Conaman^®^), afatinib (Gilotrif^®^), osimertinib (Tagrisso^®^), olmutinib (Olita^TM^), neratinib (Nerlynx^®^), dacomitinib (Vizimpro^®^), and lazertinib (Leclaza^®^) and (ii) monoclonal anti-EGFR antibodies such as cetuximab (Erbitux^®^), panitumumab (Vectibix^®^), necitumumab (Portrazza^®^), and nimotuzumab [8,9,10,11,12,13,14]. High levels of EGFR expression, phosphorylated v-akt oncogene homolog (AKT), or extracellular signal-regulated kinase (ERK) have been found in TNBC [14,15,16,17,18,19,20]. Recently, enhanced expression of proinflammatory chemokines by EGFR signaling has been suggested as a potential contributing factor to the inflammatory burden causing cancer progression and a higher mortality rate in patients with TNBC [15]. Unfortunately, targeted EGFR monotherapy in TNBC has not been successful with response rates less than 5% [3,21]. However, combination strategies of anti-EGFR therapeutics with other drugs have been suggested as promising approaches to treat TNBC [14,18,19,20,22,23,24,25,26,27].

The nuclear factor kappa light chain enhancer of activated B cells (NF-κB) is constitutively activated in most cancers, including TNBC, through various signaling pathways [28]. Activation of NF-κB is primarily regulated through its inhibitor, the inhibitor of NF-κB (IκB). Dissociation and subsequent degradation of phospho-IκB (p-IκB), which is mediated by IκB kinase (IKK) complex, leads to activation and nuclear translocation of the NF-κB transcription factor complex. In the nucleus, the NF-κB complex transactivates its target genes involved in immune regulation, anti-apoptosis, and cell proliferation [28,29,30].

The IKK complex has been demonstrated to play a crucial role in coupling inflammation and cancer [29]. The IKK complex consists of two catalytic subunits, IKKα (IKK1) and IKKβ (IKK2), and a regulatory subunit IKKγ (also known as NF-κB essential modulator, NEMO) [29]. These two catalytic subunits of IKK in humans have distinct roles in tumorigenesis; IKKβ has an NF-κB-dependent tumor-promoting functions, whereas IKKα has an NF-κB-independent role in tumor metastasis [29]. IKKα has been reported to be able to translocate into the nucleus to phosphorylate CREB-binding protein (CBP) leading to cancer development [31,32,33].

EGFR-NF-κB crosstalk has been reported in the following contexts: (i) EGFR activates NF-κB either directly or indirectly through various pathways in normal or cancer cells including human ER negative [ER(−)] breast cancer cells [34,35,36,37,38,39,40,41,42,43] and (ii) the IKK/NF-κB axis activates EGFR signaling in human cancer cells [44,45,46,47]. Increased activation of NF-κB confers EGFRi resistance [34,48,49,50,51,52]. For example, NF-κB induces the expression of anti-apoptotic proteins BCL-extra-large (BCL-XL) and BCL2-related protein A1 (BCL2A1), which confer anticancer drug resistance in a Mucin 1 carboxy-terminal subunit (MUC1-C)-dependent manner [53,54]. MUC1-C oncoprotein has been reported to be highly expressed in TNBC [55,56]. The IKK/NF-κB pathway has been suggested as a potential therapeutic target to treat several types of cancers including TNBC [31,41,50]. However, the effect of IKK inhibition on EGFRi resistance in TNBC has not been explored yet. In addition, in small cell lung cancer (SCLC) cells, NF-κB has been suggested to be a downstream target of PI3K p110α since small molecule inhibitors of p110α reduce the expression of NF-κB, resulting in decreases in the expression levels of NF-κB transcriptional targets such as BCL2 and BCL-XL in a SCLC cell line [57]. However, the EGFR-NF-κB crosstalk in TNBC has not been fully understood.

In this report, we demonstrated the potentiation of the EGFRi gefitinib by the IKK inhibitor IKK16 (also known as IKK Inhibitor VII) in human TNBC cell lines. Co-treatment with gefitinib and IKK16 (Gefitinib+IKK16) synergistically reduced viability of TNBC cells including HS578T, MDA-MB-231, and MDA-MB-468 cells. Long-term survival rates of these TNBC cells were also diminished by the Gefitinib+IKK16 treatment. The Gefitinib+IKK16 combination further inhibited the phosphorylation of the mammalian target of rapamycin (mTOR), glycogen synthase kinase-3 beta (GSK3β), and ribosomal protein S6 (RPS6) compared with the single treatments. Phosphorylation and nuclear translocation of NF-κB p65 was further inhibited, and inhibition of NF-κB transcriptional activity was enhanced by the Gefitinib+IKK16 combination. NGS analysis revealed the synergistic inhibition of NF-κB/RELA target genes and enhanced induction of potential tumor suppressors in response the Gefitinib+IKK16 combination.

## 2. Materials and Methods

### 2.1. Reagents and Cell Culture

The fifty-five protein kinase inhibitors (PKIs) were purchased from following sources: BML-275, FR 180204, IKK16, GW 843682X, NSC 109555, NU7441, PD407824, PF 573228, SB 218078, TCS PIM-1-1, TCS PIM-1-4a, and TPCA-1 from Tocris Biosciences (Bristol, UK); indirubin-3′-monoxime and Ro-31-8220 from Calbiochem (San Diego, CA, USA); A-769662, bosutinib, chelerythrine, CP690550, fasudil, gefitinib, imatinib, nilotinib, PKC412, roscovitine, SNS-314, and tozasertib from LC Laboratories (Woburn, MA, USA); AT7867, AT9283, AZD1152, AZD1480, BI 2536, BIX 02189, CHIR-99021, CI-1040, CYC116, danusertib, enzastaurin, GDC-0879, INCB018424, JNJ-7706621, KU-55933, LY2228820, MLN8237, PD-0325901, PF-4708671, PLX-4032, PLX-4720, SB216763, SNS-032, SP600125, VX-702, Y-27632, and ZM447439 from Selleck Chemicals (Houston, TX, USA); U0126 from Promega (Madison, WI, USA); TBCA from Millipore (Burlington, MA, USA).

All TNBC cells in this study were obtained from the American Type Culture Collection (Manassas, VA, USA). The cultured cells were monitor by trypan blue cell counting as described previously [58].

### 2.2. PKI Screening

A combinatorial drug screening was performed as described previously [20,22,27]. In brief, MDA-MB-231 cells (1000 cells/well) in 96-well plates were treated with gefitinib and fifty-five PKIs in a 6 × 5 concentration matrix in duplicates for 72 h [27]. The cell viability was measured by assessing cellular metabolic activity with 4 mg/mL 3-(4,5-Dimethylthiazol-2-yl)-2,5-Diphenyltetrazolium Bromide (MTT), as described previously [19,59,60]. Synergism was determined by the classification index (CI), as described previously [20,22,27] as follows: CI = (the viability with gefitinib) × (the viability with PKI)/(the viability with the gefitinib and PKI combination). CI > 1 indicates supra-additivity; CI = 1, additivity; and CI < 1, sub-additivity. The numbers of combination points with CI > 1.3 were assigned as quantitative indices of synergism.

### 2.3. Clonogenic Survival Assay

TNBC cells, in 6-well plates, were treated with indicated drugs for 24 h, cultivated for 10–14 days in normal growth media, and the colonies were stained with crystal violet dissolved in a solubilizing buffer [1:1 mixture (*v*/*v*) of 0.1 M sodium phosphate buffer (pH4.5) and ethanol] as previously described [20,22,27]. The number of colonies were determined after imaging the colonies using an image scanner.

### 2.4. Western Blot Analysis

The cells (2 × 10^5^ cells/60-mm dish) were treated with PKIs for 2 h or 24 h in normal growth media. The cells were lysed with RIPA buffer containing a protease and phosphatase inhibitor cocktail (ThermoFisher Scientific, Waltham, MA, USA). Protein concentration was determined by BCA assay kit (Thermo Fisher Scientific, Waltham, MA, USA). The following antibodies were used in this study: AKT, p-AKT (S473), ERK1/2, p-ERK1/2 (T202/Y204), GSK3β, p-GSK3β (S9), IκB, p-IκB (S32), Lamin B1, mTOR, p-mTOR (S2448), NF-κB p65, p-NF-κB p65 (S536), the 90 kDa ribosomal S6 kinase (p90RSK), p-p90RSK (S380), RPS6, p-RPS6 (S235/236), STAT3, p-STAT3 (T705), and peroxidase-conjugated secondary antibodies (anti-rabbit IgG and anti-mouse IgG) from Cell Signaling Technology (Denver, MA, USA); β-actin from Bethyl Laboratories (Montgomery, TX, USA); and β-tubulin from Sigma-Aldrich (St. Louis, MI, USA). Original blots see Supplementary File S1.

### 2.5. Reporter Gene Assay

NF-κB-Luc vectors were previously described [61]. Cells were transiently transfected with NF-κB-Luc using Lipofectamine 2000 (Invitrogen, Carlsbad, CA, USA) on a 60-mm plate. One day after transfection, the cells were re-plated on 24 well plates (2 × 10⁴ cells/well) and incubated overnight. Then the cells were treated with gefitinib, IKK16, or Gefitinib+IKK16 for 24 h. Luciferase assays were performed using a Dual-Luciferase^®^ Reporter Assay System (Promega, Madison, WI, USA) according to the manufacturer’s instructions. Data are presented as the mean ± SEM of results from at least three independent experiments performed in triplicates.

### 2.6. Next-Generation Sequencing (NGS) Analysis

MDA-MB-231 cells were treated with vehicle control, gefitinib (3 μM), IKK16 (1.5 μM), or Gefitinib+IKK16 (3 μM and 1.5 μM, respectively) for 24 h in duplicates. NGS analysis was performed by Macrogen (Seoul, Korea). In brief, total RNA was isolated and treated with DNase. Ribosomal RNA (rRNA) was removed by ribo-zero rRNA removal kit. Isolated RNAs were randomly fragmented and converted to cDNA by reverse transcription. Prepared cDNAs with adapters were amplified by polymerase chain reaction (PCR) before sequencing. The raw transcriptome data was qualified by Phred quality score, trimmed, and further analyzed. For mapping cDNA fragments, genomic reference (GRCh38) was used.

### 2.7. Quantitative Real Time Polymerase Chain Reaction (qRT-PCR) Analysis

Total RNA (1 μg) isolated from the treated cells was reverse transcribed to cDNA using PrimeScript first-strand cDNA synthesis kit (Takara Korea Biomedical Inc., Seoul, Republic of Korea) according to the manufacturer’s instructions. Amplification of each cDNA was monitored using conf (PCR Biosystems Inc., Wayne, PA, USA) on a StepOnePlus instrument (Waltham, MA, USA). Specific primers used are listed in Table S1. Data are presented as the mean ± SEM of results from at least three independent experiments performed in triplicates.

### 2.8. Statistical Analysis

At least three independent experiments were performed in triplicate. Representative data are presented as the mean ± standard deviation (SD). One-way analysis of variance (ANOVA) with a post-hoc Tukey’s honest significance difference (HSD) test was used to compare differences between groups. Differences among groups were considered statistically significant when * *p* < 0.05, ** *p* < 0.01, and *** *p* < 0.005, respectively.

## 3. Results

### 3.1. Identification of IKK16, an IKK Inhibitor, as a Potentiator of Gefitinib

**Figure 1:**
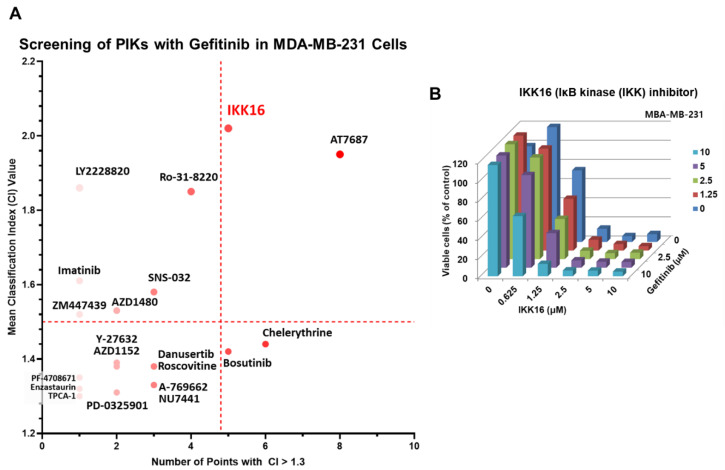
Identification of IKK16 as a synergistic partner of gefitinib for anticancer activity in MDA-MB-231 cells. (**A**) The number of points with CI values greater than 1.3 and the mean CI values for these points are depicted. IKK16 was identified as a strong potentiator of gefitinib. The dashed lines indicate the thresholds for selection (*x* = 4.8 and *y* = 1.5, respectively. CI, classification index (see Section 2). (**B**) The MTT screening results for IKK16 in combination with gefitinib in MDA-MB-231 cells.

We have performed a cell viability screening of protein kinase inhibitors (PKIs) in combination with the EGFR inhibitor gefitinib in a human TNBC cell line, MDA-MB-231. As reported previously [19,22,27,62,63,64,65,66,67,68], various PKIs targeting the phosphoinositide-3-kinase (PI3K)/AKT/mTORC1 pathway have been repeatedly identified as potentiators of gefitinib, implicating that this pathway may contribute to intrinsic resistance of TNBC to EGFRis (Figure 1 and Table 1). Among them, AT7867, an inhibitor of AKT/p70 S6 kinase (p70S6K)/protein kinase A (PKA), was previously reported as a promising PKI to potentiate anticancer activity of gefitinib in MDA-MB-231 cells [27]. In this study, we analyzed the previous screening results further, and found that our new analysis more clearly revealed the potential candidates of co-treatment with gefitinib in MDA-MB-231 cells (Figure 1A). IKK16 alone was relatively potent to reduce the viability of MDA-MB-231 cells compared with gefitinib or TPCA-1 (Figure 1A Combination of IKK16 with gefitinib resulted in a synergistic antiproliferative effect in multiple combination points (Figure 1B).

Interestingly, the protein kinase C (PKC) inhibitors, such as Ro-31-8220, chelerythrine, and enzastaurin, were identified as potential candidates for gefitinib potentiation in MDA-MB-231 cells (Figure 1 and Table 1). In addition, imatinib, which had previously been reported as a combinational treatment partner of the dual EGFR/human EGFR receptor 2 (HER2) inhibitor lapatinib [93], was also identified from the screening. Imatinib is an US FDA-approved PKI for the treatment of rare gastrointestinal cancer and acute lymphocytic leukemia [13]. Another interesting feature of the screening results is that PKIs targeting the aurora kinases (AURKs) were found to be potentiators for gefitinib. AZD1152 (barasertib), danusertib (PHA-739358), and ZM-447439 are targeting AURKs (Table 1). PKIs, targeting kinases involved in DNA damage repair, have been in the list of candidates, such as the DNA-dependent protein kinase (DNA-PK) and checkpoint kinase 1 (CHEK1). Protein kinases, such as adenosine monophosphate-activated protein kinase (AMPK), Janus kinase 2 (JAK2), and P38α were also identified as potential targets to synergize gefitinib efficacy in MDA-MB-231 cells. None of these protein kinases have been reported previously as potential targets for EGFRi potentiation [14].

Among these PKIs, we selected IKK16 for further investigation. IKK16 is a selective IKK inhibitor for IKKβ, IKK complex, and IKKα with IC_50_ values of 40, 70, and 200 nM, respectively [78]. TPCA-1 (GW683965) is also a selective inhibitor of IKKβ with an IC_50_ value of 17.9 nM [88], and the potency of TPCA-1 in combination with gefitinib was relatively low (Figure 1A). Notably, TPCA-1 has been also reported as an inhibitor of Janus kinase 1 (JAK1) with an IC_50_ value of 43.78 nM [89]. To the best of our knowledge, this is the first report on synergistic efficacy of the combination of gefitinib and an IKK inhibitor in TNBC cells.

**Figure 2:**
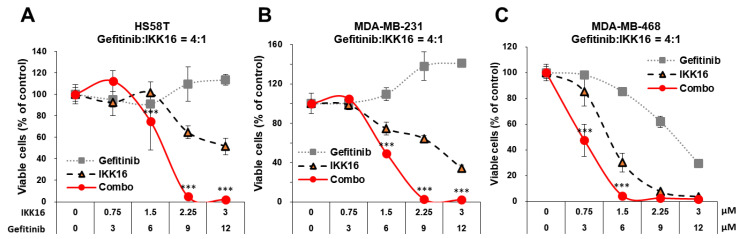
Treatment with IKK16 increased the sensitivity of TNBC cells to gefitinib. TNBC cells were treated with serially diluted concentrations of IKK16 in combination with gefitinib for 72 h. Cell viability was determined by MTT assay. (**A**–**C**) The combination effects of gefitinib with IKK16 in two MSL (HS578T and MDA-MB-231) and one BL1 (MDA-MB-468) type TNBC cells. Data are represented as the mean ± SD of results from at least three independent experiments performed in triplicates. ***, *p* < 0.001.

The synergistic effects of the Gefitinib+IKK16 combination were further accessed in two mesenchymal stem-like (MSL) cell lines, HS578T and MDA-MB-231, and a basal-like 1 (BL1) cell line, MDA-MB-468. The combination of gefitinib and IKK16 in a 4-to-1 ratio dramatically reduced the viability of all TNBC cell lines tested (Figure 2). A complete loss of viability was observed with 2.25 μM gefitinib combined with 9 μM IKK16 in HS578T and MDA-MB-231 cells, and with 1.5 μM gefitinib combined with 6 μM IKK16 in MDA-MB-468 cells. Similar to previous findings [19], the BL1 TNBC cell line MDA-MB-468 was more sensitive to gefitinib, IKK16, and their combination. Collectively, the Getifinib+IKK16 combination synergistically reduced the viability of TNBC cells in vitro.

### 3.2. Inhibition of Long-Term Survival of TNBC Cells by the Gefitinib+IKK16 Treatment

**Figure 3:**
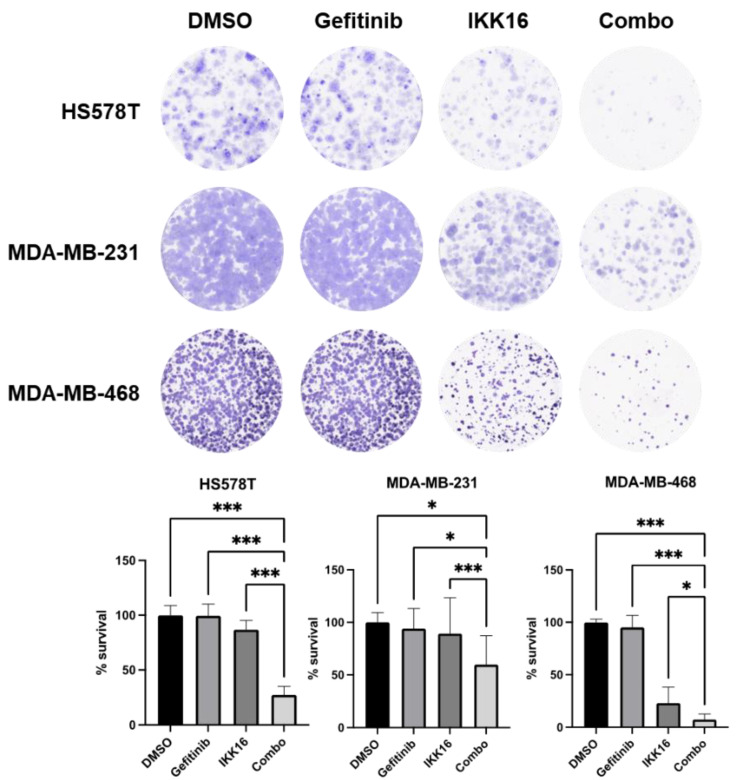
The Gefitinib+IKK16 combination reduced the survival of TNBC cells. HS578T, MDA-MB-231, and MDA-MB-468 cells were treated with 10 μM gefitinib, 2.5 μM IKK16, or the combination of 10 μM gefitinib and 2.5 μM IKK16 (Combo) for 24 h and cultivated for 10–14 days in normal growth media. The colonies were stained as described in the Materials and Methods. Representative images are shown from three independent experiments performed in triplicates. *, *p* < 0.05; ***, *p* < 0.001.

Since MTT assay results are not enough to determine the anticancer drug activity on the inhibition of proliferation and survival of residual cancer cells [94,95,96,97], we further analyzed the Gefitinib+IKK16 activity using a clonogenic assay. TNBC cells were treated with drug combinations for 24 h and further cultivated in normal growth medium without drugs for 10–14 days. As reported previously [19,20,22,27], gefitinib alone did not reduce the number of colonies from three TNBC cell lines (Figure 3). Interestingly, IKK16 itself significantly inhibited the colony formation in all TNBC cell lines tested. In addition, the survival rates of TNBC cell lines were further reduced by the Gefitinib+IKK16 treatment. Marked reduction in the colony numbers was found in HS578T and MDA-MB-468 cells.

### 3.3. Downregulation of p-STAT3, p-AKT, p-mTOR, p-GSK3β, p-RPS6 in TNBC Cells by the Gefitinib+IKK16 Treatment

**Figure 4:**
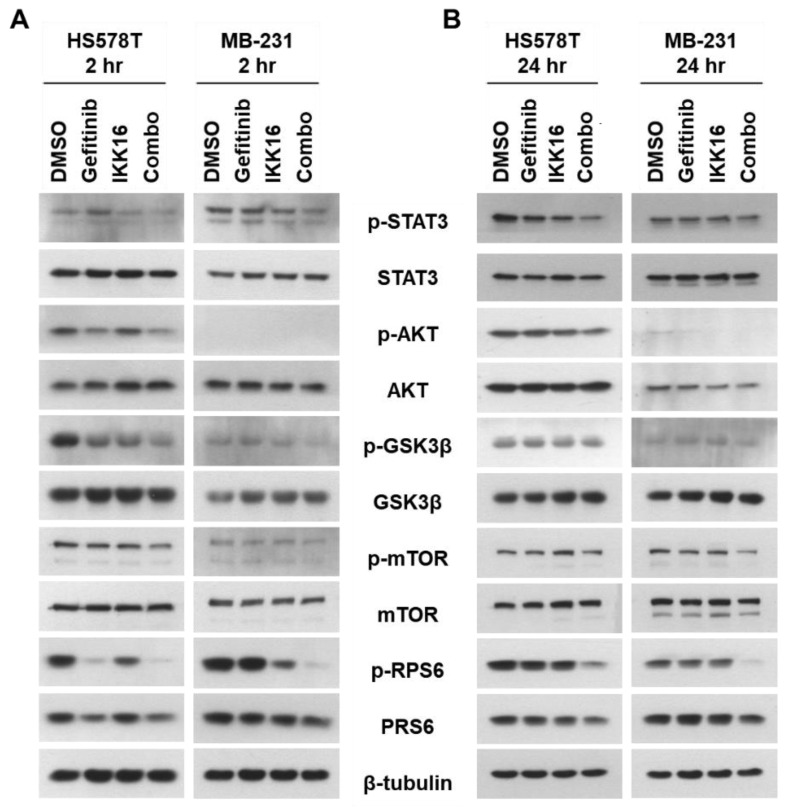
Inhibition of signaling pathways by the Gefitinib+IKK16 combination in TNBC cells. The cells were treated by drugs as indicated for 2 h (**A**) or 24 h (**B**), and the cell lysates were subjected to western blotting with antibodies against the proteins indicated. The β-tubulin was used as a loading control. Representative images were shown from three independent experiments.

Changes in the levels of signaling pathway components of interest were accessed by western blot analysis in two TNBC cell lines. HS578T and MDA-MB-231 cells were treated with drug combinations for 2 h or 24 h in normal growth media. No significant changes in the levels of p-STAT3 (Y705) in response to gefitinib treatment alone was observed (Figure 4A). Interestingly, IKK16 weakly reduced the level of p-STAT3 (Y705) in both cell lines 2 h post treatment. The Gefitinib+IKK16 combination further reduced the levels of p-STAT3 (Y705). The inhibition was pertained to 24 h (Figure 4B). To date, no previous study has reported the regulation of p-STAT3 (Y705) by IKK. The phosphorylation of tyrosine 705 residue is mediated by JAK and SRC leading to activation of STAT3 [98]. It is worthy to note that stabilization of IKKα by direct physical interaction of STAT3 has been reported to prevent its proteasomal degradation, leading to activation of non-canonical NF-κB pathway during tumorigenesis of breast epithelial cells [99].

The phosphorylation of AKT (S473) was reduced by gefitinib alone and further reduced by the Gefitinib+IKK16 combination in HS578T cells treated for 2 h. However, the gefitinib-mediated inhibition of p-AKT was abolished in HS578T cells treated for 24 h. This recurrence of p-AKT was partially suppressed by Gefitinib+IKK16 treatment in HS578T cells (Figure 4B). As previously reported, the levels of p-AKT (S473) were barely detectable in MDA-MB-231 cells cultured in normal growth media containing 10% FBS [16,17,19,20,22].

The levels of p-GSK3β (S9), the downstream target of AKT, were reduced by gefitinib alone. This inhibitory effect was more apparent in TNBC cells treated for 2 h. The Gefitinib+IKK16 combination further reduced p-GSK3β (S9) levels for up to 24 h. Interestingly, IKK16 reduced the levels of p-GSK3β (S9) in TNBC cells treated for 2 h, but not in those treated for 24 h (Figure 4B).

Treatment with gefitinib or IKK16 alone had little or no effect on the levels of p-mTOR (S2448), another AKT downstream effector. However, the Gefitinib+IKK16 treatment reduced p-mTOR (2448) levels in both cell lines. Most interestingly, the Gefitinib+IKK16 combination synergistically reduced the p-RPS6 (S235/236) levels, which were inhibited by individual treatments with both gefitinib or IKK16 in HS578T cells. The levels of total RPS6 were also marginally reduced by the Gefitinib+IKK16 treatment. It has been reported that siRNA-based knockdown of RPS6 was sufficient to reduce the viability of TNBC cells [20], suggesting RPS6 as a potential target for treating cancer [100]. Taken together, the Gefitinib+IKK16 combination suppressed the PI3K/AKT/mTOR pathway in TNBC cells.

### 3.4. Regulation of NF-κB by the Gefitinib+IKK16 Treatment in TNBC Cells

**Figure 5:**
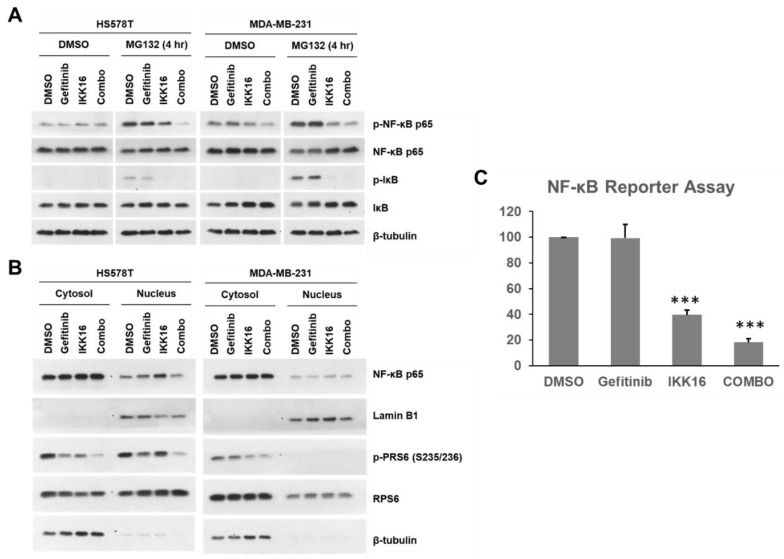
Regulation of NF-κB p65 by the Gefitinib+IKK16 treatment. (**A**) Proteasomal degradation of NF-κB p65/RelA by the Gefitinib+IKK16 treatment in the absence or presence of MG132. The cells were treated with drugs for 24 h as indicated. MG132 (10 μM) was applied for 4 h before treatment of drugs. (**B**) Subcellular localization of NF-κB p65/RelA and RPS6. The cells were treated for 24 h and the cytoplasmic and nuclear fractions were subjected to western blot analysis. Either β-tubulin or Lamin B1 was used as a loading control. Representative images were shown from three independent experiments. (**C**) NF-κB reporter gene assay. HS578T cells transfected with the NF-κB reporter gene were treated with DMSO, gefitinib 5 μM), IKK16 (1.25 μM), or Gefitinib+IKK16 (5 μM and 1.25 μM, respectively) for 24 h and luciferase activities were determined. Data from three independent experiments are shown as the mean ± SEM. ***, *p* < 0.005.

Since IKK regulates the stability of IκB and localization of NF-κB, we further assessed the changes in the levels of these proteins by western blotting. Unlike lapatinib [101], gefitinib treatment did not induce increases in p-NF-κB p65 levels (Figure 5A). As expected, the levels of IκB were increased by IKK16 treatment in HS578T and MDA-MB-231 cells (Figure 5A). Inhibition of IκB phosphorylation was evidenced in cells treated with the proteasome inhibitor MG132 [102]. Blocking IKK activities resulted in the reduction of p-NF-κB levels.

Nuclear accumulation of NF-κB p65/RelA was further evaluated in HS578T and MDA-MB-231 cells treated with IKK16 (Figure 5B). Previously, it has been reported that dephosphorylation of p-NF-κB p65 (S536) occurs in the nucleus [103]. A slight increase in NF-κB p65 was observed in cells treated with IKK16. The Gefitinib+IKK16 treatment reduced this increase in nuclear NF-κB p65 levels. However, the role of p-NF-κB p65 (S536) in tumorigenesis and cancer progression remains to be determined [104].

Unexpectedly, the extent of nuclear localization of p-RPS6 (S235/236) differed between HS578T and MDA-MB-231 cells (Figure 5B). The p-RPS6 was evenly localized in both cytoplasm and nucleus in HS578T cells, whereas an exclusively cytoplasmic localization of p-RPS6 was observed in MDA-MB-231 cells. The significance of this discrepancy remains elusive.

Since the levels of nuclear NF-κB p65 were reduced by the Gefitinib+IKK16 treatment, we further performed NF-κB reporter gene assays in HS578T cells (Figure 5C). For reporter gene assay, the concentration of EC_50_ for the Gefitinib+IKK16 treatment was selected. As expected, treatment with IKK16 alone markedly reduced the luciferase activity in HS578T cells. Little or no effect of gefitinib on the luciferase activity was observed. However, most profound reduction of the luciferase activity was achieved by the Gefitinib+IKK16 combination. Taken together, these data support that the transcriptional activity of NF-κB was synergistically reduced by the Gefitinib+IKK16 combination in HS578T cells.

### 3.5. Transcriptomic Regulation by the Gefitinib+IKK16 Treatment

**Figure 6:**
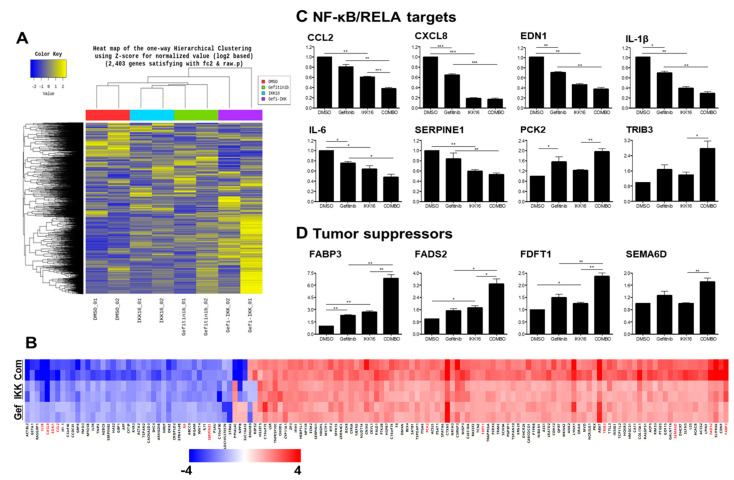
Global transcriptional changes induced by the Gefitinib+IKK16 treatment. (**A**) Heat map of a total of 2403 genes identified that showed at least 2-fold change upon the gefitinib+IKK16 treatment in duplicated samples. (**B**) Heat map of 139 genes whose mRNA levels were reproducibly modulated by the gefitinib+IKK16 combination (≤mean ± 0.15). qRT-PCR results for (**C**) NF-κB/RELA target genes and (**D**) tumor suppressor genes in MDA-MB-231 cells treated with drugs for 24 h as indicated. Data from three independent experiments are shown as the mean ± SEM. *, *p* < 0.05; **, *p* < 0.01; ***, *p* < 0.005.

The global effect on the transcriptional regulation induced by the Gefitinib+IKK16 (10 μM and 2.5 μM, respectively) combination was analyzed by NGS in MDA-MB-231 cells. A total of 2403 genes were identified that exhibited at least 2-fold change (fc2) in the duplicated samples (Figure 6A). We further narrowed down the list to 139 genes with mRNA levels reproducibly modulated by drug treatment (≤mean ± 0.15) in duplicates (Figure 6B). Twenty-three genes were identified as targets for NFKB1 or RELA by the ConsensusPathDB (http://cpdb.molgen.mpg.de/, accessed on 10 May 2022) [105] or reference analysis.

To further confirm their enhanced regulation by the Gefitinib+IKK16 combination, we performed qRT-PCR analysis of selected gene transcripts in RNA samples from MDA-MB-231 cells treated with gefitinib (10 μM), IKK16 (2.5 μM), or the Gefitinib+IKK16 combination (10 μM and 2.5 μM, respectively) for 24 h (Table 2).

In the qRT-PCR analysis, a subset of the NF-κB/RELA target genes, such as CCL2, CXCL8, EDN1, IL-1β, IL-6, and SERPINE1, was identified as targets of the Gefitinib+IKK16 treatment (Figure 6C). Generally, gefitinib alone reduced the mRNA levels of these genes, but IKK16 had more profound inhibitory effects. The Gefitinib+IKK16 combination further enhanced the gefitinib-mediated suppression of these genes. As shown in Table 2, the genes identified as targets of the combinatory treatment have important roles in tumorigenesis, and inhibition of their function induces anti-tumor effects.

Unexpectedly, the transcript levels of two RELA target genes were upregulated by the Gefitinib+IKK16 combination. One of these, PCK2, has been reported as a tumor suppressor in renal cell carcinoma [112]. The other one, TRIB3, is a master oncogenic factor. The mechanisms of transcriptional regulation of these gene have not been understood yet. In addition, it would be noteworthy that the mRNA expression of TRIB3 was markedly enhanced by the Gefitinib+IKK16 combination (Figure 6C). Although the Gefitinib+IKK16 combination showed synergistic anticancer effects, the induction of an oncogenic factor by this combination still warrants the need for further investigation of the mechanisms and/or additional drug combinations to overcome potential recurrence of drug resistance.

Interestingly, the mRNA expression levels of several potential tumor suppressor genes were markedly increased by the Gefitinib+IKK16 combination (Figure 6D). However, the underlying molecular mechanisms of this synergistic induction remain to be investigated. Taken together, the addition of IKK16 overcomes the resistance of TNBC cells to gefitinib, potentially through the transcriptional inhibition of the NF-κB/RELA target genes and induction of a set of tumor suppressor genes in TNBC cells.

## 4. Discussion

In an effort to identify potential therapeutic options to treat TNBC [14,16,17,19,20,22,27,60,100,119,120,121], we have screened small-molecule PKIs in combination with gefitinib in MDA-MB-231 cells. We found PI-103 (a DNA-PK/PI3Kα inhibitor) [19], SU11274 (a proto-oncogene c-Met [MET] inhibitor) [20], MK-2206 (an allosteric AKT inhibitor) [22], and AT7867 (an AKT/p70S6K/PKA inhibitor) [27] as potentiators of EGFRis in TNBC cells. We also found that an MAPK/ERK kinase (MEK) inhibitor, PD-0325901 (mirdametinib), further enhanced the antiproliferative and anti-clonogenic activities of the Gefitinib+AT7867 treatment in TNBC cells [27]. During these studies, we also noted that β-TrCP, RPS6, and regulatory-associated protein of mTOR (RPTOR) are potential therapeutic targets for TNBC treatment [17,20,22].

Previously, an inverse correlation has been reported in the levels of EGFR and ER in ER(-) and ER positive BC cells [122,123,124,125]. In addition, as revealed by a comparative study, EGF is a major autonomous growth-promoting factor for TNBC cells [41]. Furthermore, the level of active NF-κB in TNBC cells are elevated by the EGF-EGFR axis and inhibited by the anti-EGFR antibody and the PKC inhibitor Go6976 [41]. The activated NF-κB transactivates the cell-cycle regulator, cyclin D1, leading to increase of p-retinoblastoma (p-RB) in ER(-) cells in a PI3K/PKC/IKK-dependent manner [41]. Constitutive activation of NF-κB has also been reported in TNBCs [126,127]. Blocking activated NF-κB by small-molecule inhibitor or by expression of IκBα super-repressor leads to apoptotic cell death or reduced proliferation of TNBC cells [41,126]. More interestingly, an adaptive survival program is induced by EGFR inhibition, leading to rapid formation of the EGFR-tumor necrosis factor receptor-associated factor 2 (TRAF2)-receptor-interacting protein 1 (RIP1)-IKK complex to activate NF-κB in non-small cell lung cancer xenograft model [128].

We observed downregulation of p-NF-κB p65 in TNBC cells treated with IKK16, and this effect was further enhanced by the Gefitinib+IKK16 treatment. This result is consistent with previous results: First, both IKKα and IKKβ, the targets of IKK16, phosphorylate NF-κB p65/RelA at S536 [129,130,131,132]. Phosphorylation of NF-κB p65 (S536) has been known to inhibit its nuclear import and promote its turnover [130,133,134]. Second, AKT, the downstream effector of EGFR, phosphorylates IKKα through direct phosphorylation to activate the NF-κB p65 [135,136,137,138,139,140]. The RPTOR-dependent activation of NF-κB transcriptional activation via the mTOR-IKKα interaction suggests the positive-regulation of the IKKα/NF-κB axis by the AKT/mTORC1 pathway [141]. In addition, IKKα is also known to promote mTORC1 activation through phosphorylation of mTOR at S1415 in an AKT-dependent manner [142]. IKKα further contributes to the activation of AKT through physical association with mTORC2, leading specifically to the AKT-dependent phosphorylation and inhibition of Forkhead box O3a (FOXO3a) and GSK3β, but not other AKT targets, such as Tuberous sclerosis complex subunit 2 (TSC2) and proline-rich AKT substrate of 40 kDa (PRAS40) in several cancer cells [143].

IKK16 is a selective IKK inhibitor with IC_50_ values of 40, 70, and 200 nM for IKKβ, IKK complex, and IKKα, respectively, in cell-free enzyme assays [78]. Inhibition of NF-κB by IKK16 circumvents resistance to multitargeted RTK inhibitors, such as sunitinib and sorafenib, in renal cell carcinoma cells [48]. IKK16 also abrogates resistance of glioblastoma U87MG cells to the Gefitinib+PHA665752 (a MET inhibitor) treatment via blocking fibroblast growth factor 1 (FGF1) expression [49]. Recently, IKK16 has been reported to partially inhibit the interleukin (IL)-36β-induced p-RPS6 in CD8+ T cells [144]. Regarding this, IKKs have been revealed as an upstream stimulator of mTORC1 activation [142,145,146,147]. IKKα and mTORC1 have been reported to interact with and activate each other [141,146]. Activation of mTORC1 activates the S6 kinase (S6K) and repressed the eukaryotic translation initiation factor 4E-binding protein 1 (4E-BP1) in an AKT-dependent manner in phosphatase and Tensin homolog (PTEN)-deficient prostate cancer cells [146]. In addition, IKKβ phosphorylates and inactivates Tuberous sclerosis complex subunit 1 (TSC1), the negative regulator of mTORC1, to drive angiogenesis [145].

IKKα has been reported to activate β-catenin, whereas IKKβ downregulates β-catenin-dependent transcription [139,148]. Since IKKα activates mTORC1 [142], downregulation of β-catenin-dependent transcription might be mediated by GSK3β. In addition, the PI3K/AKT/IKKα pathway has been demonstrated to activate the β-catenin-dependent transcription [139,148,149]. However, the role of IKK-mTORC1 axis has not been explored yet in TNBC cells.

Proteosomal degradation of p-IκB is mediated by SCF-β-TrCP complex-dependent ubiquitination of IκB [28]. Interestingly, the PI3K/mTORC2 inhibition-dependent degradation of β-TrCP1 has been demonstrated to inhibit proliferation of TNBC cells [17]. Therefore, perturbation of PI3K/mTORC2 may further induce inhibition of NF-κB activation by reducing β-TrCP-dependent degradation of IκB in TNBC cells.

NGS analysis and subsequent qRT-PCR analysis suggested that blocking IKK potentiates EGFR inhibition at least partially though the suppression of NF-κB/RELA-dependent transcription of tumorigenic genes and induction of tumor suppressors. Since IKK16 also inhibits IKKα which has an NF-κB-independent role, regulation of tumor suppressor genes may be mediated by EGFR-IKKα in TNBC cells. Interestingly, IKKα-dependent activation of NOTCH1 signaling has been reported to induce p-AKT, oxidative metabolism, and transcriptional activation of survival genes in PTEN wild-type TNBC cells [150].

In summary, the present study provides evidence that supports the combined targeting of EGFR and IKK as a potential therapeutic strategy for TNBC treatment.

## 5. Conclusions

In the present study, we identified the IKK/NF-κB pathway as a potential target for enhancing the efficacy of EGFR inhibition in TNBC cells. Combined inhibition of this pathway with EGFR inhibition resulted in the reduction in cell viability and long-term survival of TNBC cells. NGS analysis revealed that a subset of the NF-κB/RELA target genes was synergistically suppressed, and expression levels of a series of tumor suppressor genes were elevated by the co-targeting EGFR and IKK with specific small molecule inhibitors. These results warrant further studies on the therapeutic potential of targeting the IKK/NF-κB pathway in combination with current therapeutics for treating TNBC in the future.

## Figures and Tables

**Table 1:** PKIs identified from the screening.

PKI	Other Names	Known Targets (IC_50_ or EC_50_ Value in nM)	Refs.
A-769662		AMPK (800)	[69]
AT7867		AKT2 (17), PKA (20), AKT1 (32), AKT3 (47), p70S6K (85)	[70]
AZD1152	Barasertib, AZD2811	AURKB (0.37)	[71]
AZD1480		JAK2 (0.26)	[72]
Bosutinib	Bosulif^®^, SKI-606, DB8	ABL1 (1), SRC (1.2)	[73,74]
Chelerythrine	-	PKC (660)	[75]
Danusertib	PHA-739358	AURKA (13), ABL1 (25), RET (31), TRKA (31), FGFR1 (47), AURKC (61), AURKB (79)	[76]
Enzastaurin	LY317615	PKCβ (6), PKCα (39), PKCγ (83), PKCε (110)	[77]
IKK 16	IKK Inhibitor VII	IKKβ/IKK2 (40), IKK complex (70), IKKα/IKK1 (200)	[78]
Imatinib	Gleevec^®^, STI571, CGP057148B	PDGFR (100), KIT (100), v-ABL (600)	[79]
LY2228820	Ralimetinib	P38α (7)	[80]
NU 7441	KU-57788	DNA-PK (14)	[81]
Ro-31-8220	Bisindolylmaleimide IX	PKCα (5), PKCβ2 (14), PKCβ1 (24), PKCε (24), PKCγ (27)	[82]
PD-0325901	Mirdametinib	MEK (0.33)	[83]
PF-4708671		p70S6K1 (160)	[84]
Roscovitine	Seliciclib, CYC202	CDK5/P35 (160)	[85]
SNS-032	BMS-387032	CDK9/Cyclin T (4)	[86]
TCS 2312		CHEK1 (60)	[87]
TPCA-1	GW683965	IKK2 (17.9), JAK1 (43.78)	[88,89]
Y-27632		ROCK1 (140; Ki); ROCK2 (300; Ki)	[90,91]
ZM-447439		AURKA (110), AURKB (130)	[92]

**Abbreviations**: ABL1, Abelson murine leukemia viral oncogene homolog 1; AKT, v-akt oncogene homolog; AMPK, adenosine monophosphate-activated protein kinase; AURKA, aurora kinase A; AURKB, aurora kinase B; AURKC, aurora kinase C; CDK, cyclin-dependent protein kinase; CHEK1, checkpoint kinase 1; DNA-PK, DNA-dependent protein kinase; FGFR1, fibroblast growth factor receptor 1; IKK, IκB kinase; JAK, Janus kinase; KIT, v-kit Hardy-Zukerman 4 feline sarcoma viral oncogene homolog; MEK, MAPK/ERK kinase; p70S6K, p70 S6 kinase; PDGFR, platelet-derived growth factor receptor; PKA, protein kinase A; PKC, protein kinase C; RET, rearranged during transfection; ROCK, Rho-associated, coiled-coil-containing protein kinase; SRC, v-src avian sarcoma (Schmidt-Ruppin A-2) viral oncogene homolog; TRKA, tropomyosin-related kinase A.

**Table 2:** Potential roles of selected genes from transcriptome analysis. ↑ upregulate; ↓ downregulate.

Gene Symbol	Description	Combo Effect	TF	Potential Roles in Cancer	Refs.
CCL2	C-C motif chemokine ligand 2	↓	NF-κB/RelA [105]	CCL2 knockdown blocks the renewal of cancer stem cells, leading to the inhibition of the progression of TNBC in vivo	[106]
CXCL8	CXC motif chemokine ligand 8	↓	NF-κB/RelA [105]	CXCL8-CXCR1/2 pathway mediates the tumorigenesis of multiple cancers, such as those of breast, prostate, lung, colon, and melanoma.	[107]
EDN1	endothelin 1	↓	NF-κB/RelA [105]	EDN1 receptor antagonist reduces migration of MCF7 breast cancer cells	[108]
IL1B	interleukin 1 beta	↓	NF-κB/RelA [105]	Upregulated in TNBC cells; Treatment of IL1 receptor antagonist decreases invasiveness of TNBC cells.	[109]
IL6	interleukin 6	↓	NF-κB/RelA [105]	Highly expressed in TNBC cellsTargeting IL6 and IL8 expression by shRNAs inhibits colony formation and survival of TNBC cells in vitro and tumor growth in vivo.	[110]
SERPINE1	serpin family E member 1	↓	NF-κB/RelA [105]	SERPINE1 knockdown reverse the paclitaxel resistance of TNBC cells by reducing vascular endothelial growth factor A (VEGFA)	[111]
PCK2	phosphoenolpyruvate carboxykinase 2, mitochondrial	↑	RelA [105]	A tumor suppressor in renal cell carcinoma	[112]
TRIB3	tribbles pseudokinase 3	↑	RelA [105]	A master oncogenic factor	[113]
FABP3	fatty acid binding protein 3	↑		A potential tumor suppressor in breast and embryonic cancers	[114,115]
FDFT1	farnesyl-diphosphate farnesyltransferase 1	↑		A potential tumor suppressor	[116]
FADS2	fatty acid desaturase 2	↑		A potential tumor suppressor	[117]
SEMA6D	semaphorin 6D	↑		A tumor suppressor in pancreatic cancer	[118]

## Data Availability

Data in this study will be available from the corresponding author upon reasonable request.

## Supplementary Materials

The following supporting information can be downloaded at: https://www.mdpi.com/article/10.3390/cancers14215215/s1, Table S1: Primers used for qRT-PCR. File S1: Original WB Images.
