# Supplementary material for: Inhibition of IκB Kinase Is a Potential Therapeutic Strategy to Circumvent Resistance to Epidermal Growth Factor Receptor Inhibition in Triple-Negative Breast Cancer Cells
Source: Cancers (Basel). 2022 Oct 24;14(21):5215. doi: 10.3390/cancers14215215 (PMC9654813; doi:10.3390/cancers14215215)
Supplement: Supplementary file 1 [file cancers-14-05215-s001.zip › cancers-1970177-supplementary materials/File S1-original wb.pdf]

Supplementary WB Images for

Inhibition of IκB Kinase is a Potential Therapeutic Strategy to Circumvent Resistance to Epidermal Growth Factor Receptor Inhibition in Triple-Negative Breast Cancer Cells

Yong Weon Yi [1,†], Kyu Sic You [1,2,†], Sanghee Han [3], Jeong-Soo Park [1], Seok-Geun Lee [3,*], and Yeon-Sun Seong [1,2,3,*]

Figure 4 – 2hr

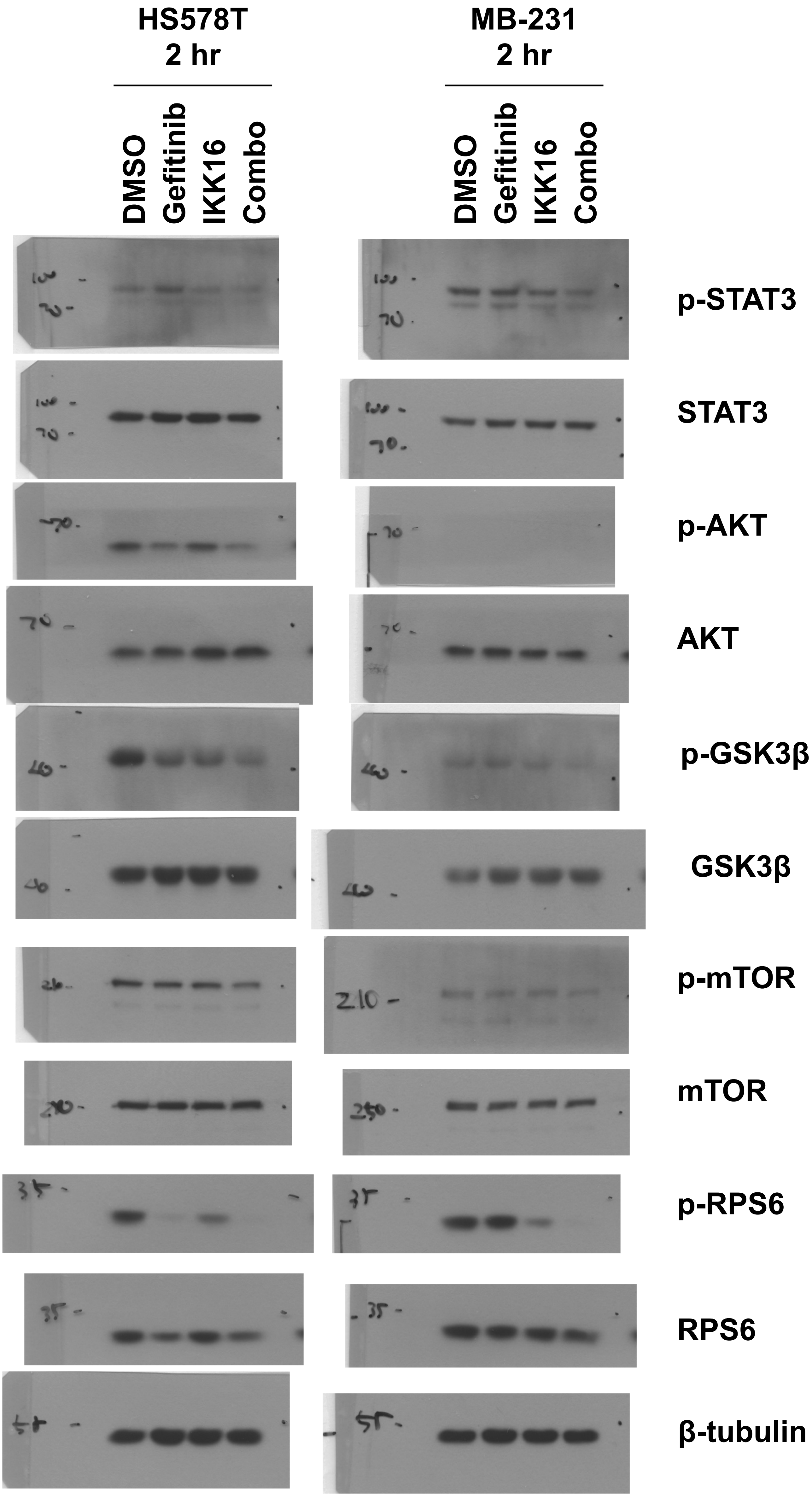
HS578T
2 hr
MB-231
2 hr
DMSO
Gefitinib
IKK16
Combo
DMSO
Gefitinib
IKK16
Combo
p-STAT3
STAT3
p-AKT
AKT
p-GSK3β
GSK3β
p-mTOR
mTOR
p-RPS6
RPS6
β-tubulin

Figure 4 – 24hr

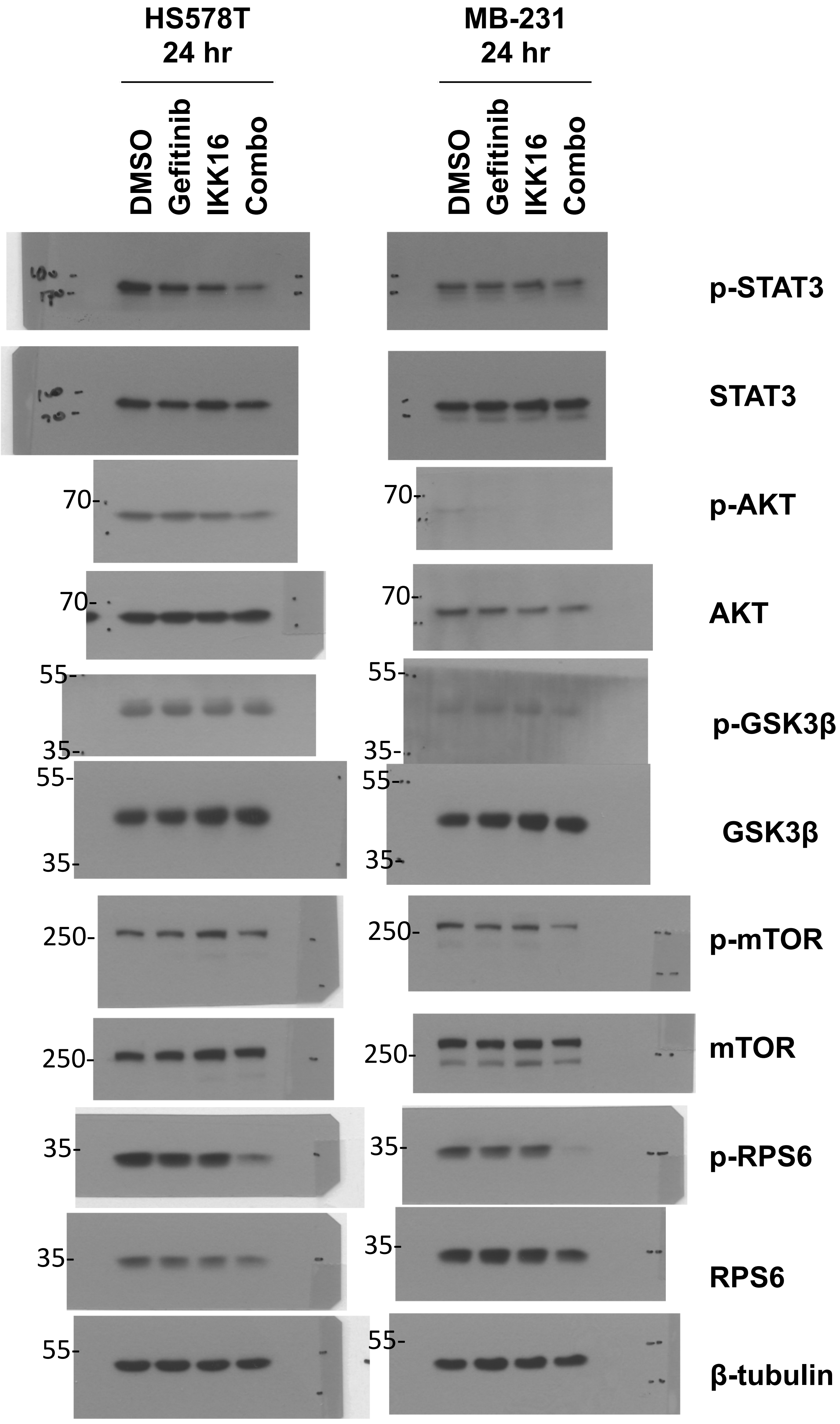

Figure 5

A

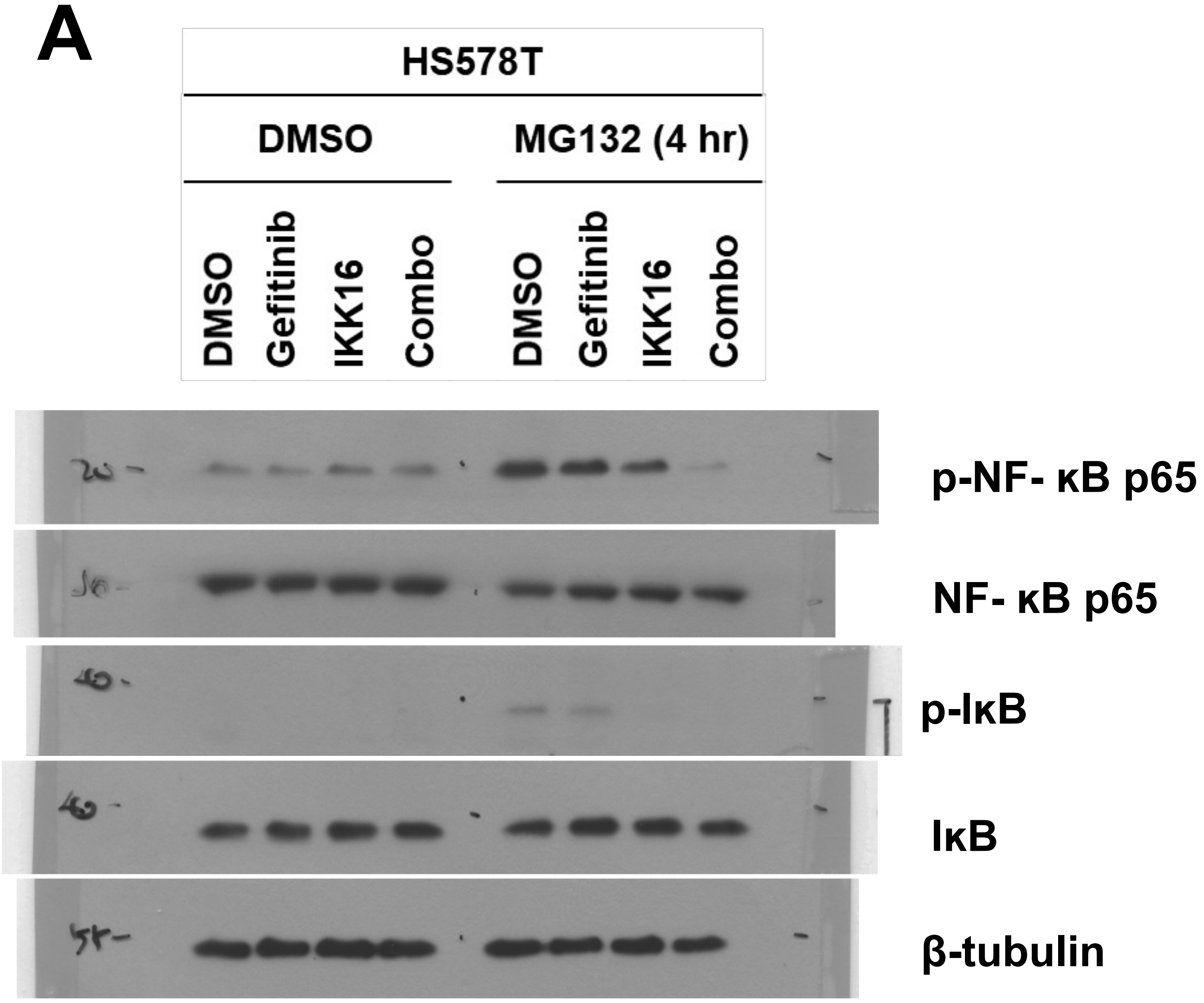
HS578T
DMSO
MG132 (4 hr)
DMSO
Gefitinib
IKK16
Combo
DMSO
Gefitinib
IKK16
Combo
p-NF- κB p65
NF- κB p65
p-IκB
IκB
β-tubulin

Figure 5

A

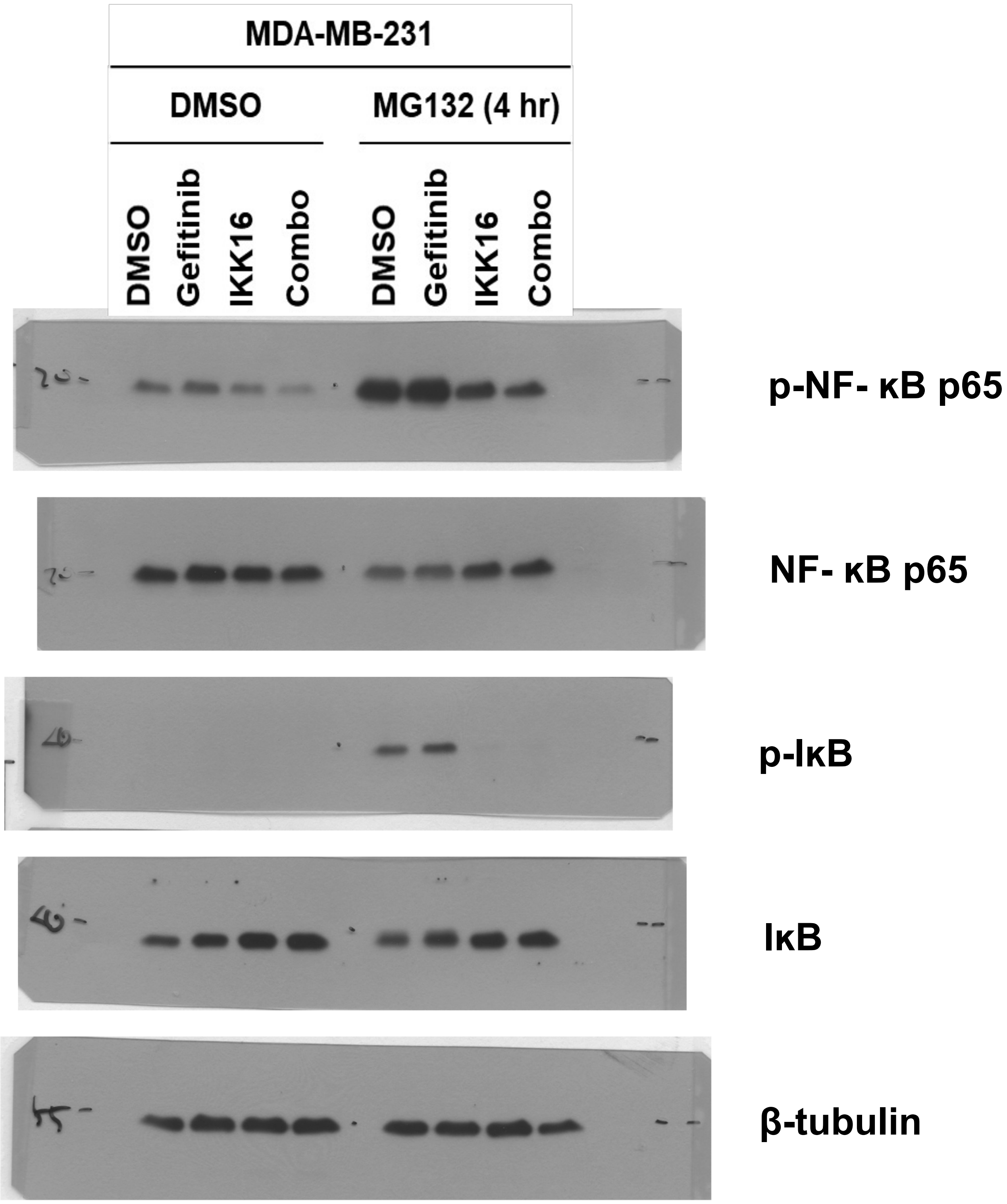
MDA-MB-231
DMSO
MG132 (4 hr)
DMSO
Gefitinib
IKK16
Combo
DMSO
Gefitinib
IKK16
Combo
p-NF- κB p65
NF- κB p65
p-IκB
IκB
β-tubulin

Figure 5

B

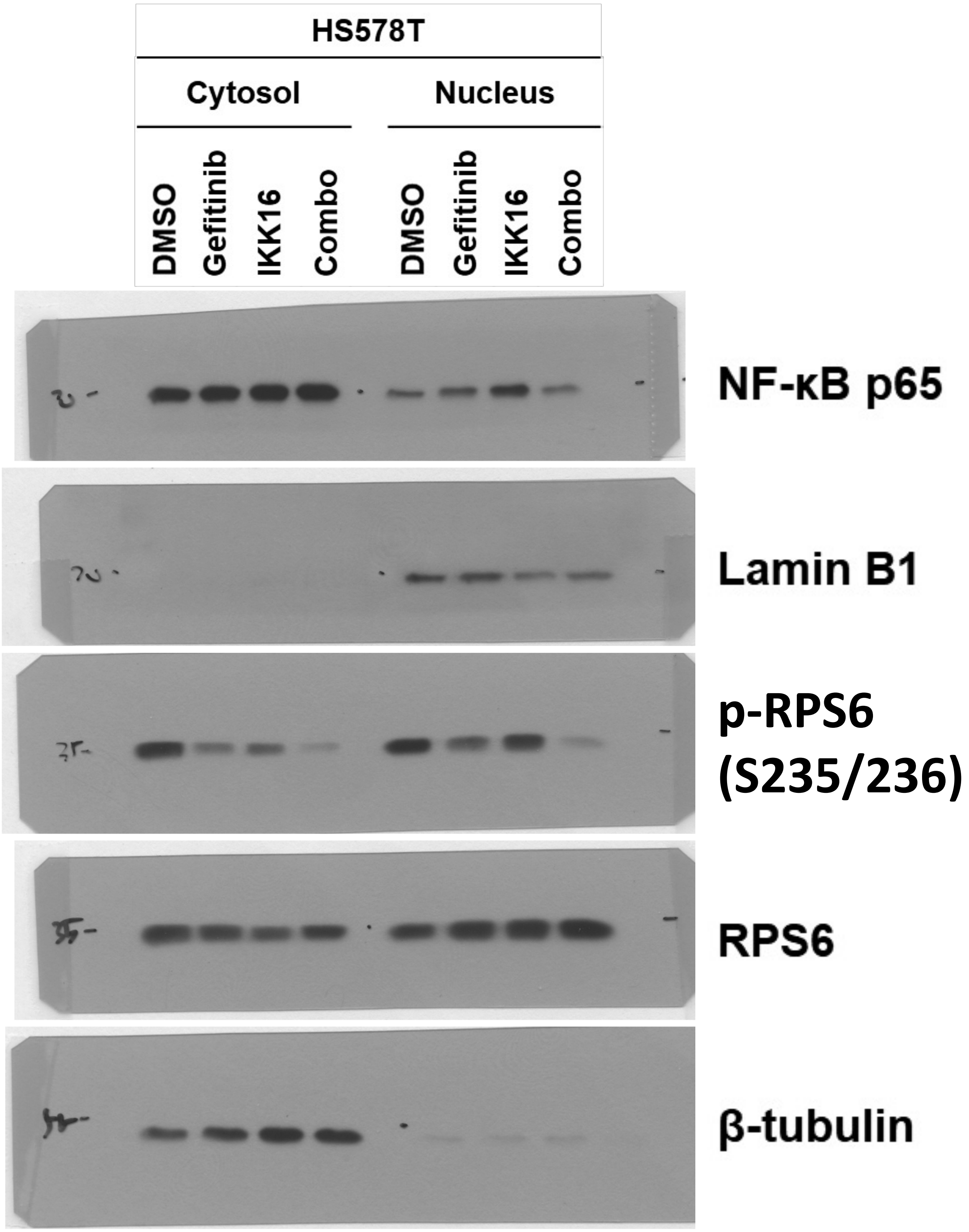
HS578T
Cytosol
Nucleus
DMSO
Gefitinib
IKK16
Combo
DMSO
Gefitinib
IKK16
Combo
NF-κB p65
Lamin B1
p-RPS6 (S235/236)
RPS6
β-tubulin

Figure 5

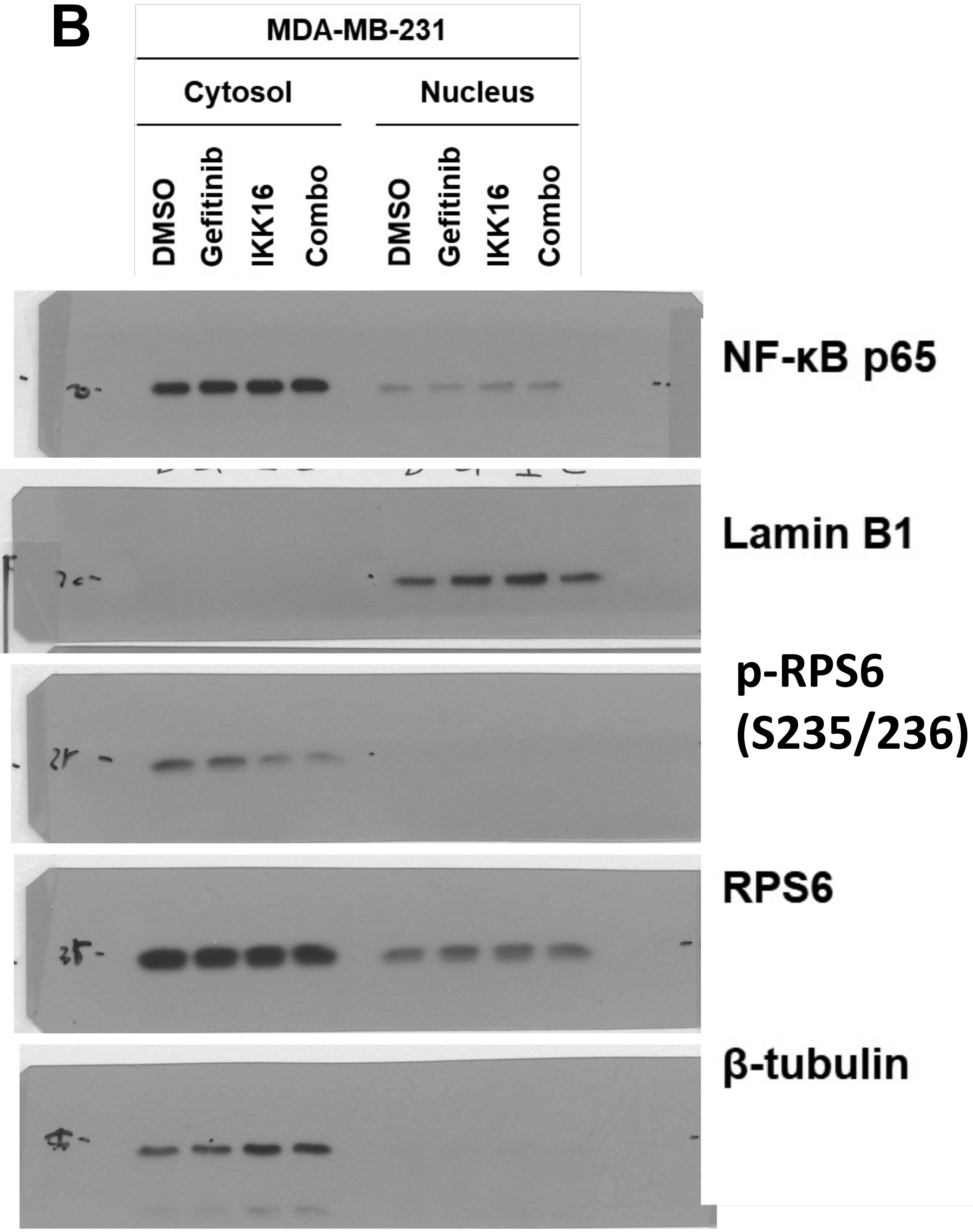
B
MDA-MB-231
Cytosol
Nucleus
DMSO
Gefitinib
IKK16
Combo
DMSO
Gefitinib
IKK16
Combo
NF-κB p65
Lamin B1
p-RPS6 (S235/236)
RPS6
β-tubulin
