# Supplementary material for: Inhibition of IκB Kinase Is a Potential Therapeutic Strategy to Circumvent Resistance to Epidermal Growth Factor Receptor Inhibition in Triple-Negative Breast Cancer Cells
Source: Cancers (Basel). 2022 Oct 24;14(21):5215. doi: 10.3390/cancers14215215 (PMC9654813; doi:10.3390/cancers14215215)
Supplement: Supplementary file 1 [file cancers-14-05215-s001.zip › cancers-1970177-supplementary materials/Table S1.pdf]

Table S1. Primers used for qRT-PCR

Gene	Forward (5′ → 3′)	Reverse (5′ → 3′)
**CCL2**	ATGAAAGTCTCTGCCGCCCTTCTGT	AGTCTTCGGAGTTTGGGTTTGCTTG
**CXCL8**	AGGGTTGCCAGATGCAATAC	GCAAACCCATTCAATTCCTG
**EDN1**	CAGCAGTCTTAGGCGCTGAG	ACTCTTTATCCATCAGGGACGAG
**IL1B**	TTAAAGCCCGCCTGACAGA	GCGAATGACAGAGGGTTTCTTAGA
**IL-6**	AGGGCTCTTCGGCAAATGTA	GAAGGAATGCCCATTAACAACAA
**SERPINE1**	GTGTTTCAGCAGGTGGCGC	CCGGAACAGCCTGAAGAAGTG
**PCK2**	CATCCGAAAGCTCCCCAAGTA	TGGAAATCAGCTGGGGACATC
**TRIB3**	AAGAAGCGGTTGGAGTTGGATGAC	GTTGCACGATCTGGAGCAGTAGG
**FABP3**	GTGGAGTTCGATGAGACAACAGC	TGGTCTCTTGCCCGTCCCATTT
**FDFT1**	GGTCCCGCTGTTACACAACT	AAAACTCTGCCATCCCAATG
**FADS2**	TCATGACCATGATCGTCCATAAGAA	GCTCCCAGGATGCCGTAGAA
**SEMA6D**	TTTCCCAGTTGAGGGCAGTC	AGGGCGTCCTCTAAAAACCG
**GAPDH**	AACAGCGACACCCACTCCTC	CATACCAGGAAATGAGCTTGACAA
